# Epigenetic age acceleration of cervical squamous cell carcinoma converged to human papillomavirus 16/18 expression, immunoactivation, and favourable prognosis

**DOI:** 10.1186/s13148-020-0822-y

**Published:** 2020-02-10

**Authors:** Xiaofan Lu, Yujie Zhou, Jialin Meng, Liyun Jiang, Jun Gao, Xiaole Fan, Yanfeng Chen, Yu Cheng, Yang Wang, Bing Zhang, Hangyu Yan, Fangrong Yan

**Affiliations:** 1 State Key Laboratory of Natural Medicines, China Pharmaceutical University, Nanjing, People’s Republic of China; 2 Research Center of Biostatistics and Computational Pharmacy, China Pharmaceutical University, Nanjing, People’s Republic of China; 3 Division of Gastroenterology and Hepatology, Key Laboratory of Gastroenterology and Hepatology, Ministry of Health, Renji Hospital, School of Medicine, Shanghai Jiao Tong University, Shanghai Institute of Digestive Disease, Shanghai, People’s Republic of China; 4 Department of Urology, The First Affiliated Hospital of Anhui Medical University; Institute of Urology & Anhui Province Key Laboratory of Genitourinary Diseases, Anhui Medical University, Hefei, Anhui People’s Republic of China; 5 Department of Urology, University of Rochester Medical Center, Rochester, NY USA; 6 School of Medicine, Nantong University, Nantong, People’s Republic of China; 7 Department of Radiology, The Affiliated Nanjing Drum Tower Hospital of Nanjing University Medical School, Nanjing, People’s Republic of China

**Keywords:** Epigenetic age acceleration, Prognosis, Human papillomavirus, Immunoactivation, Cervical squamous cell carcinoma

## Abstract

**Background:**

Ageing-associated molecular changes have been assumed to trigger malignant transformations and the epigenetic clock, and the DNA methylation age has been shown to be highly correlated with chronological age. However, the associations between the epigenetic clock and cervical squamous cell carcinoma (CSCC) prognosis, other molecular characteristics, and clinicopathological features have not been systematically investigated. To this end, we computed the DNA methylation (DNAm) age of 252 CSCC patients and 200 normal samples from TCGA and three external cohorts by using the Horvath clock model. We characterized the differences in human papillomavirus (HPV) 16/18 expression, pathway activity, genomic alteration, and chemosensitivity between two DNAm age subgroups. We then used Cox proportional hazards regression and restricted cubic spline (RCS) analysis to assess the prognostic value of epigenetic acceleration.

**Results:**

DNAm age was significantly associated with chronological age, but it was differentiated between tumour and normal tissue (*P* < 0.001). Two DNAm age groups, i.e*.* DNAmAge-ACC and DNAmAge-DEC, were identified; the former had high expression of the E6/E7 oncoproteins of HPV16/18 (*P* < 0.05), an immunoactive phenotype (all FDRs < 0.05 in enrichment analysis), CpG island hypermethylation (*P* < 0.001), and lower mutation load (*P* = 0.011), including for *TP53* (*P* = 0.002). When adjusted for chronological age and tumour stage, every 10-year increase in DNAm age was associated with a 12% decrease in fatality (HR 0.88, 95% CI 0.78–0.99, *P* = 0.03); DNAmAge-ACC had a 41% lower mortality risk and 47% lower progression rate than DNAmAge-DEC and was more likely to benefit from chemotherapy. RCS revealed a positive non-linear association between DNAm age and both mortality and progression risk (both, *P* < 0.05).

**Conclusions:**

DNAm age is an independent predictor of CSCC prognosis. Better prognosis, overexpression of HPV E6/E7 oncoproteins, and higher enrichment of immune signatures were observed in DNAmAge-ACC tumours.

## Background

There are 528,000 new cervical cancer cases and 266,000 deaths due to cervical cancer worldwide each year, which exceeds the values of any other gynaecological tumour [1]. Squamous cells are involved in 80 to 90% of cervical cancer cases, of which approximately 95% are caused by persistent infection with carcinogenic human papillomavirus (HPV) [2], one of the most common sexually transmitted diseases in both men and women worldwide [3].

Ageing-associated molecular change has been assumed to trigger malignant transformations since previous reports recognized age as a strong demographic risk factor for cervical cancer [4, 5]. Molecular predictive models have proposed measuring human chronological and biological age with epigenetic DNA methylation (DNAm) [6–9]; among these models, a multiple tissue age predictor, the “Horvath’s clock”, was found to be strongly correlated with chronological age [10]. Epigenetic age acceleration, which is the vertical shift between DNAm age and chronologic age, has been reported to be tightly associated with clinical outcomes of many diseases [11, 12], including tumours with prognostic effects varying across cancer types [13–15]. Puzzlingly, two studies focusing on breast cancer drew opposite conclusions regarding epigenetic clock-based prognostic effects. Ren J et al. reported that decreased DNAm age is associated with poor prognosis after adjusting for major clinical variables, including tumour stage and oestrogen receptor status [16], while Kresovich J et al. found that DNA methylation-based measures of biological age acceleration are significantly associated with increased risk of developing breast cancer [17]. These studies suggested an essential role for biological age in tumour development and progression.

Although a previous study showed that the DNAm age of 195 endocervical adenocarcinoma patients was not significantly associated with overall survival [18], the prognostic value of the epigenetic clock in cervical squamous cell carcinoma (CSCC) remains yet unexplored. In this study, we focused on CSCC and comprehensively examined associations of DNAm age with clinical outcomes, tumour clinicopathological features, and molecular characteristics in CSCC patients.

## Methods

### Study population

Molecular data of patients diagnosed with cervical cancer were obtained from TCGA. Methylation data assessed by TCGA using Infinium 450 K arrays were downloaded from Xena Public Data Hubs (https://xena.ucsc.edu/public-hubs), which includes 307 tumour samples and 3 adjacent-normal samples. Due to the limited normal tissue, another 197 normal cervical samples with detailed chronological age information were retrieved from three external GEO datasets (GSE20080 [9], GSE30758 [19] and GSE30759 [20]) and were profiled by Illumina Infinium 27 K arrays. To be specific, GSE20080 contains 30 normal cervical samples based on cervical smears; GSE30758 comprises 152 normal cervical samples that were drawn from a randomized trial in a primary cervical screening population; GSE30759 includes 15 normal cervical samples that were collected from women who underwent a hysterectomy for uterine fibroids. Transcriptome HTSeq-counts data of the TCGA-CESC project were downloaded from the Genomic Data Commons using R package “*TCGAbiolinks*”; this repository includes 304 tumour samples and 3 adjacent-normal samples, among which 252 tumours diagnosed with cervical squamous cell carcinoma were selected for the purpose of this study. Corresponding patient survival and clinicopathological information were also retrieved. The raw, paired-end reads in FASTQ were also obtained for virus detection. Somatic mutation data were downloaded from TCGA PanCanAtlas and filtered for CESC tumour type.

### DNA methylation age and epigenetic age acceleration

DNA methylation age, based on Horvath’s clock model, was calculated from the methylation β values using the agep() embedded in R package “*wateRmelon*” [21]. Briefly, the Horvath’s clock model utilizes β values of 353 CpG loci to calculate DNAm age based on the formula of DNAmAge = *inverse*. *F*(*α*_0_ + *α*_1_ × *CpG*_1_ + … + *α*_353_*CpG*_353_) where *F* is a function for age transformation and *α*_*i*_ is the coefficient obtained from the elastic net regression model. Epigenetic age acceleration (i.e. vertical shift) was then calculated by extracting chronological age from DNAm age individually. Tests for DMPs were carried out in the R package “*ChAMP*” via the champ.DMP() function with default parameters [22]. Probes were determined to gain methylation if the difference was statistically significant (FDR < 0.05) and greater than 0 between two groups. More stringent hypermethylated probes were considered if the average methylation level was greater than 0.3 in the treatment group but less than 0.2 in the control group with FDR less than 0.05, and vice versa for hypomethylated probes. Methylation β matrix was first filtered and imputed, and then normalized through the embedded BMIQ method by default. The methylation heatmap was presented with M values for its stronger signals.

### Data pre-processing for transcriptome HTSeq-counts

Ensembl ID for protein-coding mRNAs was annotated to the Symbol name by GENCODE27. We calculated the number of fragments per kilobase of non-overlapped exon per million fragments mapped (FPKM) first and subsequently transferred FPKM into transcripts per kilobase million (TPM) values. Those mRNAs with TPM less than 1 in over 90% samples were considered noise and removed from downstream analysis.

### Virus detection from RNA-Seq

The VirusSeq [23] algorithm was harnessed to computationally subtract human sequences and generate a set of nonhuman sequences (e.g. viruses) in RNA-Seq. We aligned the RNA-Seq libraries to both human and HPV genomes to quantify the host and viral gene expression and determine the HPV status. Among all tumour samples, 168 HPV16, 38 HPV18, 1 HPV26, 1 HPV30, 7 HPV31, 8 HPV33, 2 HPV34, 6 HPV35, 22 HPV45, 1 HPV51, 8 HPV52, 1 HPV56, 7 HPV58, 3 HPV59, 7 HPV68, 1 HPV69, and 2 HPV70 were identified and 21 samples remained no infection. Seven oncoproteins of HPV—i.e. E1, E2, E5, E6, E7, L1, and L2—were quantified as FPKM values. A positive integration event is a fusion candidate having at least four discordant read pairs and at least one junction spanning read, and if a tumour sample contained at least one such event, positive genic integration was considered [23].

### Molecular characterization of two DNA methylation age groups

We utilized the R package “*DESeq2*” to perform differential expression analysis with the standard comparison mode between the DNAmAge-ACC and DNAmAge-DEC groups [24]. The Benjamini-Hochberg procedure embedded in the package was implemented to adjust nominal *P* values (FDR) for multiple testing. Gene set enrichment analysis (GSEA) was performed on mRNA expression profiling using the R package “*clusterProfiler*” [25, 26]. The presence of infiltrating immune/stromal cells in tumours was estimated by the R package “*ESTIMATE”* [27]. Mutation landscape was analysed by the R package “*maftools*” with removal of 100 FLAGS genes first [28, 29].

### Prediction of chemosensitivity

Base on the largest publicly available pharmacogenomics database (GDSC, the Genomics of Drug Sensitivity in Cancer, https://www.cancerrxgene.org/), we employed the R package “*pRRophetic*” to predict the chemotherapeutic sensitivity for each tumour sample; the estimated IC_50_ of each treated with specific chemotherapy drug was obtained by ridge regression, and prediction accuracy was measured through 10-fold cross-validation with the GDSC training set. Default values were selected for all parameters, including “combat” for removal of batch effect, “allSoldTumours” for tissue type, and mean value for summarizing duplicate gene expression [30].

### Statistical analyses

All statistical analyses were conducted in R (Version: 3.5.2) using a Fisher’s exact test for categorical data and a two-sample Mann-Whitney test or Student’s *t* test for continuous data when appropriate. Correlation between two continuous variables was measured by Pearson’s correlation coefficient. Fisher’s *r*-to-*z* transformation was used to calculate a value of *z* that was applied to assess the significance of the difference between two correlation coefficients. Survival analysis was performed using the R package “*survival*”. Specifically, a Kaplan-Meier curve was generated for survival rates of patients with difference detection of a log-rank test. A Cox proportional hazards regression model adjusted for confounding clinical variables was used to calculate HRs and 95% CIs for epigenetic age status regarding both OS and PFS. The RCS analysis was used in the multivariate Cox proportional hazard model to explore the association between continuous epigenetic age acceleration level and patient clinical outcomes with equally spaced knots based on the R packages “*Hmisc*” and “*smoothHR*” [31], where the optimal number of knot was determined by minimizing the model’s Akaike information criterion. For all statistical analysis, a two-tailed *P* value less than 0.05 was considered statistically significant.

## Results

### Differential correlation between DNAm age and chronological age in normal and tumour tissues

**Fig. 1:**
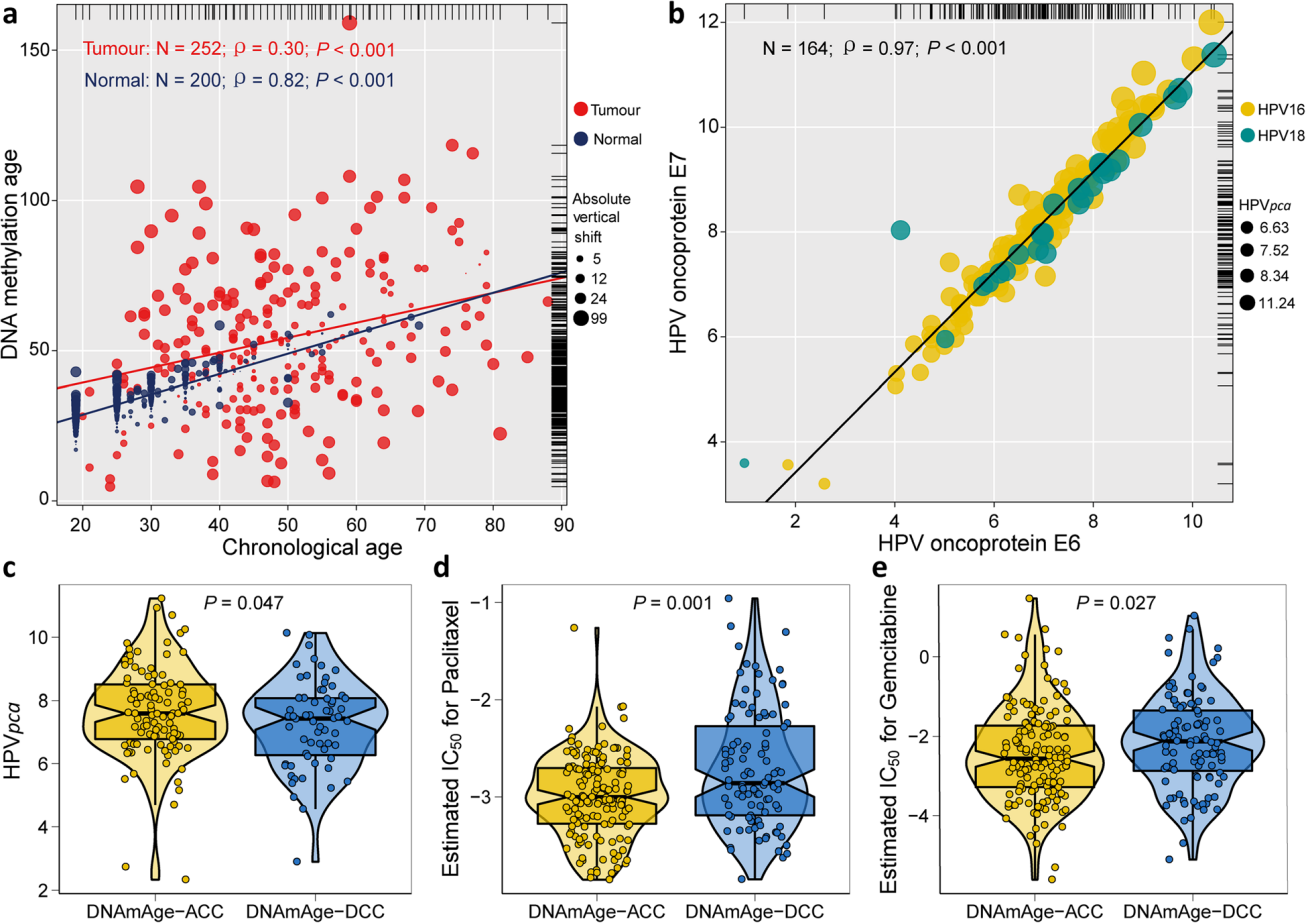
Correlations between DNAm age and chronological age and other molecular characteristics of DNAm age groups. **a** DNAm age of 200 mixed normal cervical samples predicts chronological age with a decent correlation coefficient, whereas such correlation was much weaker in 252 tumour samples from TCGA. **b** HPV oncoproteins E6 and E7 were highly correlated in 164 samples infected with two high-risk virus types—HPV16/18. The marginal rug line describes the distribution of the continuous variable at the corresponding coordinate axis. **c** DNAmAge-ACC group presented with significantly higher *HPV*_*pca*_ scores than DNAmAge-DEC group and was inferred to be much more sensitive to two commonly used chemotherapy drugs, i.e. paclitaxel and gemcitabine, as shown in (**d**) and (**e**), respectively. The test for association between paired samples used Pearson’s correlation coefficient. Two-tailed statistical *P* values were calculated by a two-sample Mann-Whitney test or Student’s *t* test when appropriate

The study population included 252 tumour samples retrieved from The Cancer Genome Atlas (TCGA) and a total of 200 normal cervical samples as a control from both the TCGA and Gene Expression Omnibus (GEO) data. Among the 200 normal samples, chronological age is highly correlated with DNAm age (ρ = 0.82). However, this correlation is largely missing in tumour tissue (ρ = 0.30), which indicates that the pattern of DNA methylation observed in normal cervical tissue is disrupted in cervical tumour tissue (*P* < 0.001; Fig. 1a). Patients were further dichotomized to either the epigenetic age-accelerated or age-decelerated group according to the individual vertical shift between DNAm age and chronological age. They were classified as ‘DNAmAge-ACC’ or age accelerated if the shift was greater than zero, and they were classified as ‘DNAmAge-DEC’ or age decelerated if the shift was less than zero.

### Association between DNAm age and HPV16 and 18 expression

Similar to our previous study [32], we calculated a comprehensive variable *HPV*_*pca*_ by principal component analysis (PCA) based on HPV E6 and E7 oncoproteins because the joint action of the two oncoproteins is required for HPV-induced malignancy [33]. Two common HPV types with high risk, i.e. HPV16 and HPV18, were selected. As expected, E6 and E7 were strongly correlated (ρ = 0.97, *P* < 0.001; Fig. 1b), and the first component accounted for nearly all variation (99.2%). Univariate Cox proportional hazards regression revealed that increased *HPV*_*pca*_ was associated with better overall survival (OS) with a hazard ratio (HR) of 0.89 and 95% confidence interval (CI) of 0.80 to 0.99 (*P* = 0.038), but it was not associated with progression-free survival (PFS) (not shown). Tumours infected with either HPV16 or HPV18 presented with significantly different *HPV*_*pca*_ levels regarding DNAm age group (*P* = 0.047; Fig. 1c). We then examined whether a difference existed in HPV-integration events between the DNAm age groups, and no significant difference was observed (not shown).

### Characteristics and the relationships with DNAm age in CSCC

The distributions of patients’ body mass index (BMI) (divided according to World Health Organization (WHO) categories), race, ethnicity, and clinical stage were not significantly different between the DNAmAge-ACC and DNAmAge-DEC groups (Table 1). However, patients with DNAmAge-ACC tumours had more tumour-free cases (*P* = 0.02) and had better response to their first treatment (*P* = 0.007), which was consistent with favourable prognosis. Interestingly, we also found the DNAmAge-ACC group was enriched with premenopausal cases (*P* = 0.004), and a greater likelihood of HPV negativity existed in the DNAmAge-DEC group (*P* = 0.018). To investigate whether the DNAmAge-ACC group was more sensitive to treatment (e.g. chemotherapies), we constructed a predictive model on three commonly used chemo drugs (i.e. cisplatin, paclitaxel, and gemcitabine) and confirmed that the DNAmAge-ACC group was significantly more likely to respond to paclitaxel (*P* = 0.001; Fig. 1d) and gemcitabine (*P* = 0.027; Fig. 1e).

**Table 1:** Demographic and clinicopathological characteristic and the associations with DNAm age for 252 CSCC patients

Characteristics	Total (%)	Epigenetic age (0-year cut-off)		*P*^a^
		DNAmAge-ACC	DNAmAge-DEC	
		(*N* = 148)	(*N* = 104)	
Age (years)				
Continuous^b^	252 (100)	45.6 ± 12.9	53.5 ± 15.5	< 0.001
Category (median)				
< 47	122 (48)	89 (60)	33 (32)	< 0.001
≥ 47	130 (52)	59 (40)	71 (68)	
BMI (WHO)				0.763
< 18.5	9 (4)	6 (4)	3 (3)	
18.5–25	74 (29)	43 (29)	31 (30)	
25–30	56 (22)	31 (21)	25 (24)	
> 30	72 (29)	46 (31)	26 (25)	
Missing	41 (16)	22 (15)	19 (18)	
Race				1.000
White	169 (67)	99 (67)	70 (67)	
Others	52 (21)	31 (21)	21 (20)	
Missing	31 (12)	18 (12)	13 (13)	
Ethnicity				0.222
Hispanic or Latino	20 (8)	15 (10)	5 (5)	
Not Hispanic or Latino	140 (56)	81 (55)	59 (57)	
Missing	92 (36)	52 (35)	40 (38)	
Stage				0.878
I + II	187 (74)	112 (76)	75 (72)	
III + IV	57 (23)	33 (22)	24 (23)	
Missing	8 (3)	3 (2)	5 (5)	
Tumor status				0.025
With tumor	68 (27)	34 (23)	34 (33)	
Tumor free	161 (64)	107 (72)	54 (52)	
Missing	23 (9)	7 (5)	16 (15)	
Treatment outcome^c^				0.007
Response	150 (60)	98 (66)	52 (50)	
Non-response	25 (10)	9 (6)	16 (15)	
Missing	77 (30)	41 (28)	36 (35)	
Menopause				0.004
Post	70 (28)	31 (21)	39 (38)	
Pre	96 (38)	64 (43)	32 (31)	
Missing	86 (34)	53 (36)	33 (31)	
HPV				0.018
Positive	238 (94)	144 (98)	94 (90)	
Negative	10 (4)	2 (1)	8 (8)	
Missing	4 (2)	2 (1)	2 (2)	

^a^Fisher’s exact test for categorical data and a two-sample Mann-Whitney test for continuous data

^b^Continuous values are represented with mean ± standard deviation

^c^Response means complete remission/response partial and remission/response; non-response means progressive disease and stable disease

### Co-occurrence of epigenetic age acceleration, immunoactivation, and hypermethylation

**Fig. 2:**
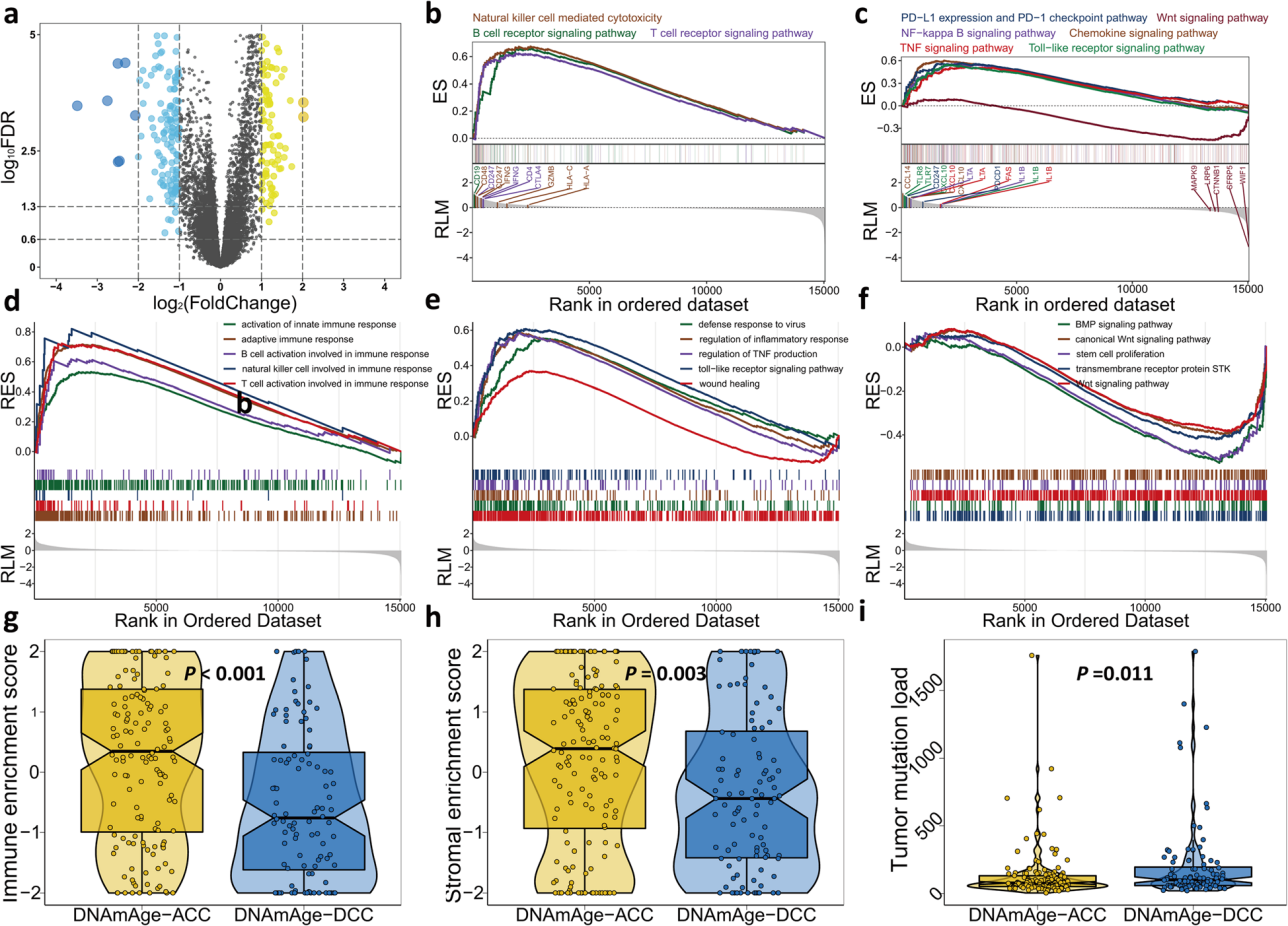
Molecular characteristics of two DNAm age groups. **a** Volcano plot of differentially expressed genes for DNAmAge-ACC group compared with the DNAmAge-DEC group with 103 upregulated and 184 downregulated genes. GSEA identified upregulated **b** lymphocyte-related pathways and **c** other immune-related signalling pathways. GSEA further identified upregulated **d** immune response functions and **e** other functions related to immunoactivation for DNAmAge-ACC group, whereas **f** BMP signalling pathway, serine/threonine kinase signalling pathway and Wnt signalling pathway were enriched for the DNAmAge-DEC group. There was a significantly higher presence of infiltrating immune/stromal cells but a lower tumour mutation load in the DNAmAge-ACC group compared with DNAmAge-DEC group, as shown in (**g**), (**h**), and (**i**), respectively. ES: enrichment scores; RES: running enrichment scores; RLM: ranked list metric. Two-tailed statistical *P* values were calculated by a two-sample Mann-Whitney test or Student’s *t* test when appropriate

To further elucidate molecular differences associated with DNAm age, we performed differential expression analysis and identified 103 and 184 significantly upregulated genes (fold change > 2, false discovery rate (FDR) < 0.05) for the DNAm-ACC and DNAm-DEC groups, respectively (Fig. 2a, Additional file 1: Table S1). Notably, we found that immune- and proliferation-related terms were specifically enriched in the DNAm-ACC and DNAm-DEC groups, respectively (Additional file 1: Table S2). For example, upregulated genes in the DNAm-ACC group were enriched for immune-related terms, such as activation of innate immune response (FDR < 0.001), interferon-alpha production (FDR = 0.003) and T cell differentiation (FDR = 0.015); upregulated genes in the DNAm-DEC group demonstrated enrichment in stem cell proliferation (*P* = 0.005, FDR = 0.062). Enrichment analysis further uncovered that patients with epigenetic age acceleration presented a universal immunoactive pattern reflected in upregulated lymphocyte pathways (i.e. T cells, B cells, and natural killer cells; all, FDR < 0.01; Fig. 2b, Additional file 1: Table S3), tumour necrosis factor (TNF) signalling (FDR = 0.002; Fig. 2c, Additional file 1: Table S3), immune response (all, FDR < 0.01; Fig. 2d, Additional file 1: Table S4), and other functions related to immunoactivation (i.e. wound healing, defence response to viruses; Fig. 2e). The DNAmAge-DEC group was enriched with the bone morphogenic protein (BMP) signalling pathway, serine/threonine kinase signalling pathway and Wingless-type (Wnt) signalling pathway (all, FDR < 0.01; Fig. 2c, f, Additional file 1: Table S4). As expected, the DNAmAge-ACC group showed a significantly higher presence of infiltrating immune/stromal cells compared with the DNAmAge-DEC group (*P* < 0.001 for immune and *P* = 0.004 for stromal; Fig. 2g, h).

**Fig. 3:**
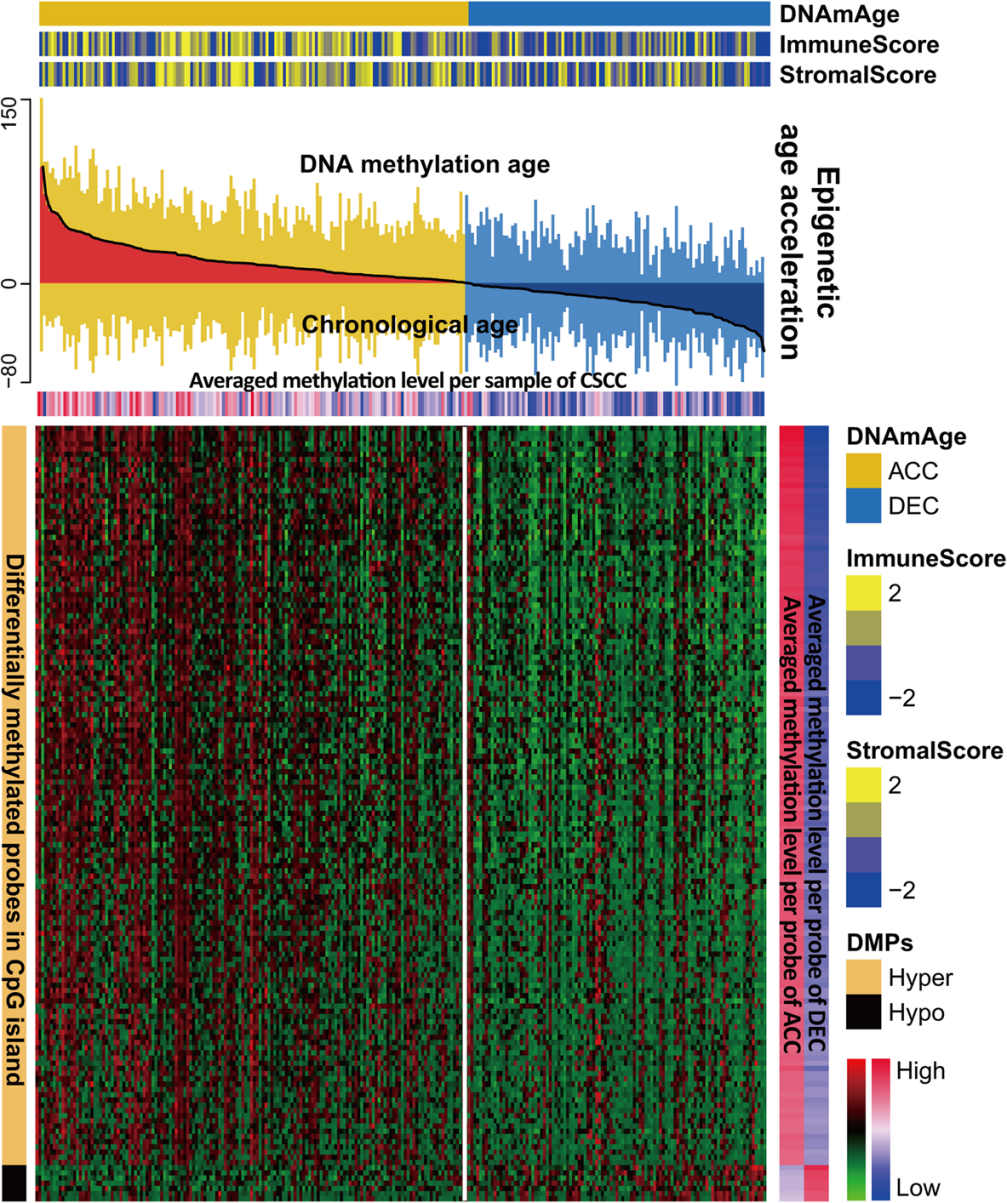
Differential DNA methylation pattern in CpG islands between two DNAm age groups. A total of 142 stringent hypermethylated probes and 7 hypomethylated probes were identified for the DNAmAge-ACC group. The heatmap based on DNA methylation M values demonstrates a co-occurrence of epigenetic age acceleration and immunoactivation as well as CpG island hypermethylation. DMPs: differentially methylated probes

We further linked our previously defined two HPV subtypes of CSCC [32] and found that the HPV16-IMM subtype was highly enriched in the DNAmAge-ACC group (*P* < 0.001). Furthermore, among genes that were mutated in more than 10 samples, we found that the DNAm-DEC group presented a significantly higher mutation load (*P* = 0.011; Fig. 2i) and was enriched with *TP53* mutation (*P* = 0.002, FDR = 0.053; Additional file 1: Table S5). Differential methylation analysis identified 9,164 differentially methylated probes (DMPs) located in CpG islands (*P* < 0.05, FDR < 0.05; Additional file 1: Table S6), among which 8,197 (89%) probes gained methylation in the DNAmAge-ACC group while only 967 (11%) were identified for patients with decelerated age. We selected 142 more stringent hypermethylated probes and 7 hypomethylated probes for the DNAmAge-ACC group and found that the epigenetic age acceleration was significantly associated with the DNA methylation level at CpG islands (ρ = 0.53, *P* < 0.001; Fig. 3).

Although a different pattern of CpG island methylation existed between the two DNAmAge groups, a subset of samples showed reversed methylation profiles in both groups. Specifically, abnormal samples in the DNAmAge-ACC group could be those with hypomethylation profiles (averaged methylation level less than zero) and those with hypermethylation profiles (averaged methylation level greater than zero) in the DNAmAge-DEC group (Fig. 3). We analysed the potential differences in clinicopathological characteristics for the DNAm-ACC and DNAm-DEC groups for these abnormal samples. We found that patients with hypomethylation patterns in the DNAm-ACC group are significantly younger than those with hypermethylation patterns (mean ± standard deviation (SD): 41.9 ± 11.3 vs. 47.7 ± 13.4; *P* = 0.024; Additional file 1: Table S7). Since patients in the DNAm-ACC group were significantly younger than those in DNAm-DEC group (45.6 ± 12.9 vs. 53.5 ± 15.5; *P* < 0.001; Table 1), we questioned whether such differences were biased by abnormal samples with significantly younger chronological age; thus, we further compared the chronological age of hypermethylated samples in the DNAm-ACC group with those in the DNAm-DEC group, and they were significantly different (47.7 ± 13.4 vs. 53.5 ± 15.5; *P* < 0.001). Additionally, premenopausal events were enriched in abnormal samples in the DNAm-ACC group (*P* = 0.038). We did not observe significant differences in other clinical features or overall survival (*P* > 0.05, not shown).

### Association between DNAm age and clinical outcomes

**Fig. 4:**
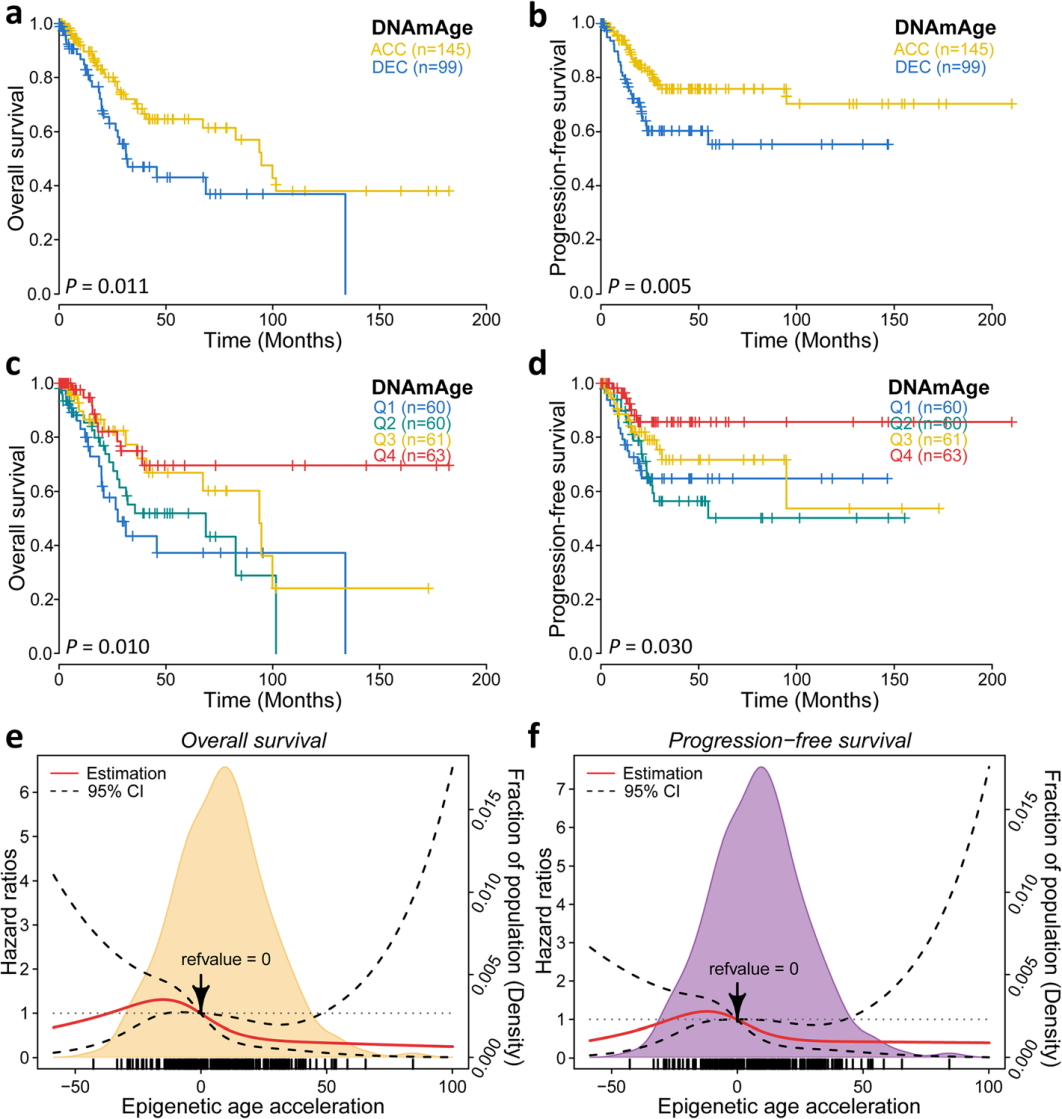
Prognostic value of epigenetic age acceleration in CSCC. Patients belonging to DNAmAge-ACC showed significantly favourable prognosis despite of **a** OS or **b** PFS by Kaplan-Meier estimator with log-rank test. DNAm age was further discretized into quartiles: Q1 (4.8, 38.3), Q2 (38.3, 51.5), Q3 (51.5, 67.5), Q4 (67.5, 159), and rates of **c** OS and **d** PFS were also well distinguished. The association between epigenetic age acceleration and the prognosis of CSCC when adjusted for chronological age (binary) and tumour stage was presented with cubic spline graphs of the adjusted HR (solid red line) and 95% CI (dotted black line). Knots: − 33.5 (5th), − 4.1 (35th), 13.1 (65th), and 44.4 (95th) of the distribution of epigenetic age acceleration; Reference value 0, which means that DNAm age equals chronological age. Associations regarding OS and PFS are shown in (**e**) and (**f**), respectively

We then investigated whether epigenetic age acceleration was associated with patients’ clinical outcomes and found that those in the DNAmAge-ACC group had significantly more favourable prognosis despite OS (*P* = 0.011) and PFS (*P* = 0.005) according to the the Kaplan-Meier estimator log-rank test (Fig. 4a, b). Different survival rates could also be observed when using quartile DNAm age (OS: *P* = 0.010, PFS: *P* = 0.030; Fig. 4c, d). When considering OS as an outcome, every 10-year increase in DNAm age was associated with a 12% decrease in fatality when the model is adjusted for chronological age and tumour clinical stage (HR 0.88, 95% CI 0.78–0.99, *P* = 0.028, Table 2). Compared with the first quartile, the fourth quartile (HR 0.32, 95% CI 0.14–0.74, *P* = 0.008) was significantly correlated with longer survival. Additionally, the DNAmAge-ACC group had a 41% lower risk of fatality compared with the DNAmAge-DEC group when considering other covariables (HR 0.59, 95% CI 0.35–1.00, *P* = 0.050). When considering PFS as an outcome, the fourth quartile was also associated with longer PFS in the categorical analysis (HR 0.35, 95% CI 0.14–0.88, *P* = 0.025), and the DNAmAge-ACC group had a better PFS compared with the DNAmAge-DEC group (HR 0.53, 95% CI 0.30–0.93, *P* = 0.027). To further examine the relationship between continuous epigenetic age acceleration and clinical outcomes, a restricted cubic spline (RCS) model was utilized with 4 knots. It showed non-linear association between the level of acceleration and patient prognosis when adjusted for major clinical variables. Zero was chosen as the reference, which means that the DNAm age equals the chronological age, and the HRs of both fatality and progression related to acceleration level rose a little and then descended sharply and steadily when acceleration was over 30 (*P* = 0.004 for OS, *P* = 0.014 for PFS; Fig. 4e, f).

**Table 2:** Association of overall and progression-free survival with DNAm age

DNAm age	Total^a^	Overall survival		Progression-free survival	
		HR (95% CI)^b^	*P*	HR (95% CI)^b^	*P*
Continuous (per 10 years)	244	0.88 (0.78–0.99)	0.03	0.90 (0.80–1.01)	0.09
Categorical (quartile)^c^					
First	60	1.00 (ref)	–	1.00 (ref)	–
Second	60	0.85 (0.44–1.62)	0.61	1.07 (0.54–2.09)	0.85
Third	61	0.60 (0.28–1.26)	0.17	0.84 (0.39–1.81)	0.65
Fourth	63	0.32 (0.14–0.74)	0.008	0.35 (0.14–0.88)	0.03
Categorical (binary)					
Decelerated	145	1.00 (ref)	–	1.00 (ref)	–
Accelerated	99	0.59 (0.35–1.00)	0.05	0.53 (0.30–0.93)	0.03

### Sensitivity analyses

Taking zero as a cut-off to measure the difference of continuous variables is mathematically logical and straightforward, but the small difference (e.g. ± 0.5 years) between epigenetic age and chronological age may lack efficiency biologically; therefore, to strengthen our conclusions, we set a more stringent cut-off of 5 years to define the DNAmAge group. In this context, two new DNAmAge groups, that is DNAmAge-ACC* and DNAmAge-DEC*, were identified with sample sizes of 123 and 84, respectively.

**Fig. 5:**
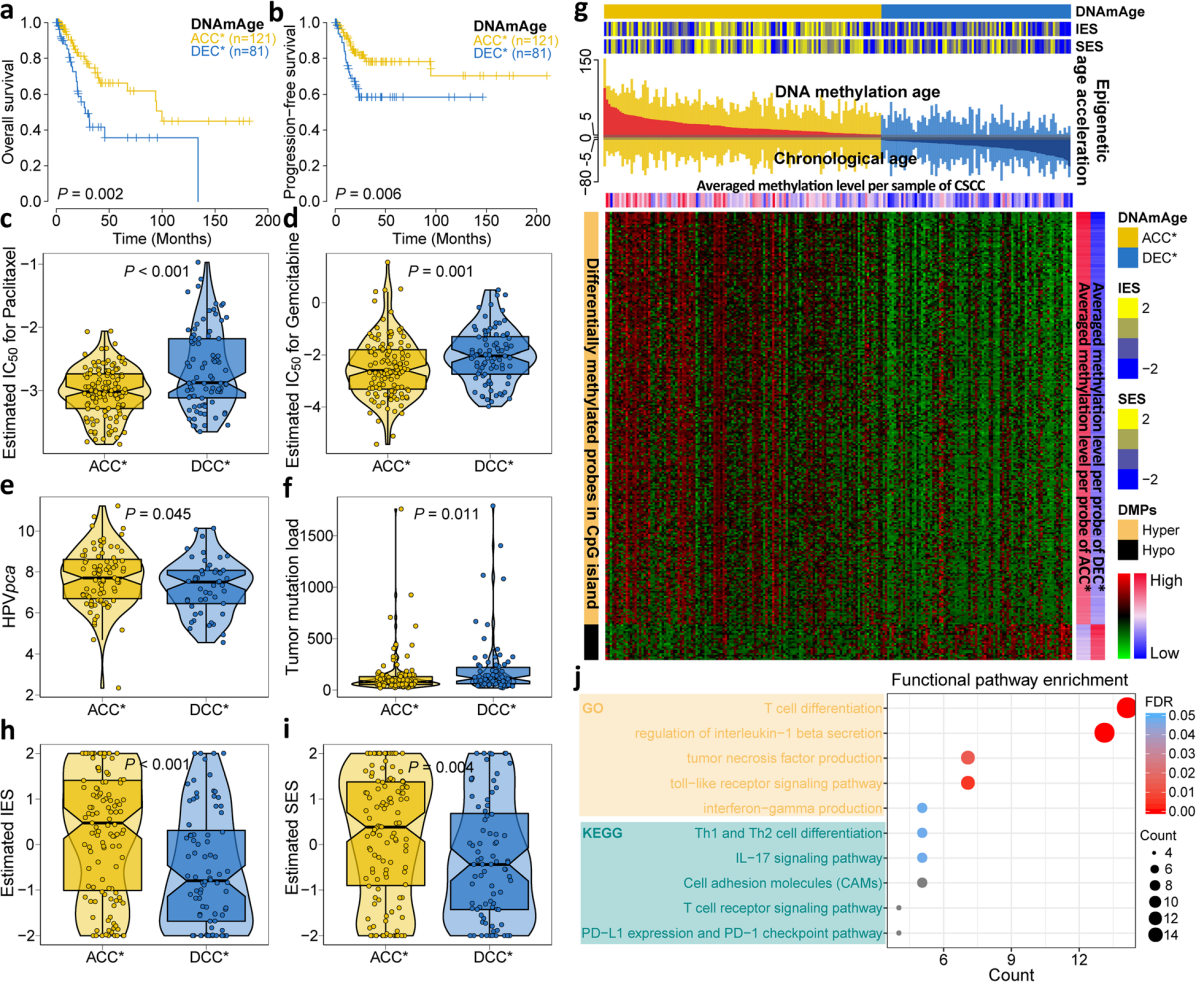
Sensitivity analyses based on new defined DNAmAge groups (i.e. DNAmAge-ACC* and DNAmAge-DEC*) with a 5-year cut-off. Patients belonging to DNAmAge-ACC* group showed significantly favourable prognosis despite **a** OS or **b** PFS by Kaplan-Meier estimator with log-rank test. The DNAmAge-ACC* group was predicted to have a higher likelihood of responding to paclitaxel and gemcitabine than DNAmAge-DEC* group, which is shown in (**c**) and (**d**), respectively; DNAmAge-ACC* presented with significantly higher *HPV*_*pca*_ scores and lower tumour mutation load than DNAmAge-DEC* group in (**e**) and (**f**), respectively. **g** Heatmap showing the differentially methylated probes (DMPs) that included a total of 246 stringent hypermethylated probes and 21 hypomethylated probes identified for DNAmAge-ACC* group. An immunoactivation phenotype was identified for DNAmAge-ACC* group due to the higher level of **h** immune enrichment score (IES) and **i** stromal enrichment score (SES) compared with the DNAmAge-DEC* group, and **j** dot plot showed immune-related GO terms and KEGG signalling pathways that were significantly enriched in DNAmAge-ACC* group

First, the Kaplan-Meier estimator confirmed a significant survival difference between the DNAmAge-ACC* and DNAmAge-DEC* groups for both OS (*P* = 0.002; Fig. 5a) and PFS (*P* = 0.006; Fig. 5b); the DNAmAge-DEC* group showed a higher risk of fatality (HR 2.41, 95% CI 1.34–4.34, *P* = 0.003) and progression (HR 2.25, 95% CI 1.24–4.08, *P* = 0.007) compared with the DNAmAge-ACC* group. Similar to our previously identified DNAmAge groups based on the 0-year cut-off, DNAmAge-ACC* tumours showed more tumour-free cases (*P* = 0.014; Additional file 1: Table S8) and better response to initial treatment (*P* = 0.017; Additional file 1: Table S8), which converged to significantly higher sensitivity to two chemotherapeutic drugs (paclitaxel: *P* < 0.001, gemcitabine: *P* = 0.001; Fig. 5c, d). We also found that the DNAmAge-ACC* group was enriched with premenopausal cases (*P* = 0.036; Additional file 1: Table S8), and the DNAmAge-DEC* group was enriched with HPV-negative cases (*P* = 0.003; Additional file 1: Table S8) along with significantly lower level of *HPV*_*pca*_ (*P* = 0.045; Fig. 5e).

We then investigated genomic and epigenetic alterations in the new DNAmAge groups. Likewise, we found that the DNAmAge-DEC* group harboured a significantly higher burden of tumour mutations (*P* = 0.011; Fig. 5f), including *TP53* (*P* = 0.004, FDR = 0.070; Additional file 1: Table S9). Differential methylation analysis identified 246 stringent hypermethylated probes and only 21 hypomethylated probes for the DNAmAge-ACC* group (Additional file 1: Table S10); the epigenetic age acceleration was significantly associated with CpG island methylation (ρ = 0.55, *P* < 0.001), which indicated a co-occurrence of epigenetic age acceleration and CpG island hypermethylation (Fig. 5g). We further profiled the immunologic landscape of the new DNAmAge groups, and similarly, the DNAmAge-ACC* group had significantly more infiltrating immune/stromal cells compared with the DNAmAge-DEC* group (*P* < 0.001 for immune and *P* = 0.004 for stromal; Fig. 5h, i). More specifically, DNAmAge-ACC* had upregulated immune-related functional pathways (all, *P* < 0.05, FDR < 0.25; Fig. 5j), whereas DNAmAge-DEC* was enriched with the Wnt (GO:0030111: regulation of Wnt signalling pathway, *P* = 0.002, FDR = 0.032; hsa04310: Wnt signalling pathway, *P* = 0.005, FDR = 0.158) and BMP signalling pathway (GO:0030509: BMP signalling pathway, *P* < 0.001, FDR = 0.007).

Again, we found that DNAm age was tightly associated with clinical outcome of CSCC. Better prognosis, overexpression of HPV, and higher enrichment of immune signatures were observed in epigenetic age-accelerated tumours.

## Discussion

In 2019, approximately 13,170 new cases of cervical cancer and 4,250 deaths due to cervical cancer were estimated to occur in the USA [34]; such high mortality and substantial heterogeneity pose an urgent need to discover effective prognostic markers for cervical cancer and CSCC. Accumulating evidence suggests epigenetic age acceleration as a novel biomarker for cancer risk [13, 15, 35]; thus, in this study, we aimed to systematically examine the association of epigenetic age acceleration with prognostic value and other molecular features in CSCC.

In the present study, a younger DNAm age was associated with worse prognosis for CSCC, which is contrary to previous findings that younger DNAm age in normal tissue is associated with improved health [36–39]. Such findings were reasonable for normal tissue because accelerated DNAm age—meaning that the individual older biologically than chronologically—is often induced by unhealthy lifestyles, susceptible heredity, harmful environmental exposures, or other stochastic events, while this might not be the case for cancerous tissue. Notably, carcinogenesis is an evolutionary process with sequential steps, including somatic mutations, subclonal evolution, and formation of cancer stem cells which possess the ability to proliferate and propagate [40, 41]. Similar to normal cells, the DNAm age of cancer cells increases with propagation, which indicates that cancer cells with lower DNAm age have higher potential to proliferate and thus might grow into more aggressive tumours [10]. This might explain why the DNAmAge-DEC group featured high proliferation and worse prognosis. Moreover, increased mutation load and enrichment of *TP53* mutation in the DNAmAge-DEC group were consistent with the association between lower DNAm age in cancer cells and higher rates of genetic mutation, including *TP53* [10].

The relationship between DNAm age and overall survival differs across tumour-derived organs [18], which suggests that increased DNAm age may be a double-edged sword with various effects in different cancers. While proliferation and cancerization may be prevented in ageing cells, chromosomal changes are likely to trigger other mutations which might lead to cancer progression and worse outcomes. In the present analysis, with confounding factors adjusted, the association between DNAm age and CSCC prognosis was consistent. Specifically, we found that patients diagnosed with CSCC had significantly favourable prognosis if epigenetic age acceleration appeared, but the mechanism underly this association remains unknown.

Persistent infection with high-risk HPV subtype leads to integration of HPV into the host genome, resulting in overexpression of oncoproteins E6 and E7, complex formation of the latter with retinoblastoma which releases transcription factor *E2F*, *E2F* binding to *DNMT1*, and eventual hypermethylation of CpG islands [42]. Badal V et al. elaborated that hypermethylation in cervical cancer suppresses malignancy in normal tissues whereas hypomethylation could promote carcinogenesis [43, 44]; the same conclusion regarding hypomethylation was reported in another study that compared normal with tumour cervical samples [45]. Furthermore, ageing-related signalling pathways—such as cell cycle, cell-cell adhesion, apoptotic, and other cell signalling pathways—are affected by hypermethylation of HPV in cervical cancer [46]. Consistent with these reports, we found higher expression of the HPV16/18 oncoproteins E6/E7 in the DNAmAge-ACC group as well as the co-occurrence hypermethylation in CpG islands.

Additionally, we also found DNAmAge-ACC group presented an immunoactive phenotype, featuring significant enrichment for immune signatures, such as T cells, B cells, natural killer cells, and inflammatory and immune response, and this group was predicted to be more sensitive to chemotherapies. DNAmAge-ACC inhibited more enriched TNF signalling, which is concordant with previous findings that enhanced TNF pathway activity promote HPV E6/E7 expression [47]. The inflammatory/immune response towards HPV was diluted when HPV shifts from the initial episomal form to an integrated transcribed form [48], but we did not observe significantly higher HPV-integration events in the DNAmAge-DEC group, which might be due to the relatively small sample size. However, the immunoactivation phenotype of the DNAmAge-ACC group could be relevant, as a subtype with higher HPV E6 activity presented higher immune response in squamous carcinomas [32, 48]. Therefore, we postulated a synergistic effect to explain why age accelerated patients generally have better prognosis in CSCC. On the one hand, HPV-triggered CpG island hypermethylation may partially cause epigenetic age acceleration; on the other hand, the highly expressed HPV16/18 in the DNAm-ACC group might stimulate a stronger inflammatory/immune response and lead to better prognosis. Moreover, the enrichment analysis indicated that serine/threonine kinase (e.g. BMP) and Wnt pathway receptors may play roles in the DNAmAge-DEC group. Previous studies have shown that BMP7 contributes to cervical tumour growth arrest [49] and Wnt/β-catenin signalling activation is involved in the development and invasion of CSCC [50, 51]. However, the roles of BMP and Wnt pathways in ageing-associated DNAm alterations are unknown and need to be investigated in the future.

We would like to acknowledge the limitations of the present investigation. Our study has a drawback of retrospective design with selection bias, and it is also limited to one centre that provided tumour samples. Additionally, we simply mixed truly normal cervical tissue samples with three adjacent-normal samples collected from TCGA in our normal cohort (*n* = 200). Due to the lack of sufficient adjacent-normal samples (*n* = 3), we cannot definitively confirm whether there is a difference between normal and adjacent-normal tissue. These findings need confirmation from other centres and larger prospective studies for their generalizability.

## Conclusions

Taken together, our findings add to the emerging literature examining the role of epigenetics in cervical cancer phenotype, which is expected to promote the prognosis of cervical cancer with epigenetic age acceleration. We demonstrated the prognostic value of DNAm age acceleration in risk stratification of CSCC patients, providing clues for future research into the mechanism of age-associated DNAm patterns in CSCC and potential therapeutic targets in CSCC treatment. Furthermore, validation for our results in large prospective cohort studies is warranted.

## Supplementary information

**Additional file 1: Table S1.** Differential expression analysis by DESeq2 between the DNAmAge-ACC and DNAmAge-DEC groups; **Table S2.** GO analysis based on differentially expressed genes; **Table S3.** KEGG GSEA analysis based on the pre-ranked gene list derived from DESeq2 result; **Table S4.** GO GSEA analysis based on the pre-ranked gene list derived from DESeq2 result; **Table S5.** Independent test between frequently mutated genes (> 10%) and DNAm age groups; **Table S6.** Differentially methylated probes in CpG islands identified by ChAMP between two DNAm age groups; **Table S7.** Demographic and clinicopathological characteristic comparison between samples with reversed methylation level in two DNAmAge groups; **Table S8.** Demographic and clinicopathological characteristic comparison between the two new DNAmAge groups; **Table S9.** Independent test between frequently mutated genes (> 10%) and new DNAm age groups; **Table S10.** Differentially methylated probes in CpG islands identified by ChAMP between two new DNAm age groups.

## Data Availability

Raw data for this study were generated at TCGA with cancer type of CESC and GEO database with Series ID of GSE20080, GSE30758 and GSE30759. Derived data supporting the findings are available from the corresponding author [F.Y.] on reasonable request.

## Abbreviations

BMP: Bone morphogenic protein
CI: Confidence interval
CSCC: Cervical squamous cell carcinoma
DMP: Differentially methylated probe
DNAm: DNA methylation
FDR: False discovery rate
GSEA: Gene set enrichment analysis
HPV: Human papillomavirus
HR: Hazard ratio
OS: Overall survival
PFS: Progression-free survival
RCS: Restricted cubic spline
TNF: Tumor necrosis factor
TPM: Per kilobase million
Wnt: Wingless-type
